# Video elicited physiological signal dataset considering indoor temperature factors

**DOI:** 10.3389/fnins.2023.1180407

**Published:** 2023-06-02

**Authors:** Kunxia Wang, Zihao Zhao, Xueting Shen, Takashi Yamauchi

**Affiliations:** ^1^School of Electronic and Information Engineering, Anhui Jianzhu University, Hefei, China; ^2^Department of Psychological and Brain Sciences, Texas A&M University, College Station, TX, United States

**Keywords:** indoor temperature stimuli, emotion recognition, physiological signals, video stimulus, classification model

## Abstract

**Introduction:**

Human emotions vary with temperature factors. However, most studies on emotion recognition based on physiological signals overlook the influence of temperature factors. This article proposes a video induced physiological signal dataset (VEPT) that considers indoor temperature factors to explore the impact of different indoor temperature factors on emotions.

**Methods:**

This database contains skin current response (GSR) data obtained from 25 subjects at three different indoor temperatures. We selected 25 video clips and 3 temperatures (hot, comfortable, and cold) as motivational materials. Using SVM, LSTM, and ACRNN classification methods, sentiment classification is performed on data under three indoor temperatures to analyze the impact of different temperatures on sentiment.

**Results:**

The recognition rate of emotion classification under three different indoor temperatures showed that anger and fear had the best recognition effect among the five emotions under hot temperatures, while joy had the worst recognition effect. At a comfortable temperature, joy and calmness have the best recognition effect among the five emotions, while fear and sadness have the worst recognition effect. In cold temperatures, sadness and fear have the best recognition effect among the five emotions, while anger and joy have the worst recognition effect.

**Discussion:**

This article uses classification to recognize emotions from physiological signals under the three temperatures mentioned above. By comparing the recognition rates of different emotions at three different temperatures, it was found that positive emotions are enhanced at comfortable temperatures, while negative emotions are enhanced at hot and cold temperatures. The experimental results indicate that there is a certain correlation between indoor temperature and physiological emotions.

## 1. Introduction

Currently, many scholars conduct research on emotion recognition around facial expressions (Singh and Nasoz, 2020; Zhang et al., 2020), speech (Wang et al., 2020; Wani et al., 2021), body postures (Piana et al., 2016), and texts (Makiuchi et al., 2021; Braunschweiler et al., 2022). The modalities of these emotional studies mentioned above are all about people’s subjective consciousness and easy to disguise. Emotion recognition using physiological signals can solve the above problems (Joesph et al., 2020). Physiological signals are generated when the human body’s internal nervous system and human tissues carry out physiological activities. These generated physiological signals are real, obvious, and objective, which are not affected by skin color, race and culture.

In psychological research, researchers have found that temperature can affect people’s emotions (Boix-Vilella et al., 2021; Cai et al., 2021). Meteorological psychologists pointed out that temperature affects people’s body, spirit, speech and behavior (Dijksterhuis et al., 2005; Kopec, 2018). Keller et al. (2005) found that warmer temperature in spring improved mood and memory compared to cooler temperature in winter. Noelke et al. (2016) found that hot temperature significantly reduced positive emotions and increased negative emotions compared with average temperature. Bushman’s study (Bushman et al., 2005) found that warmer temperature can make people nervous and irritable. Anderson et al. (2000) pointed out that high temperature increases anger and hostility, reduces vigilance and energy, and increases aggression and violence, while cold temperature shows the exact opposite effect. Some researchers (Tsai et al., 2010; Gbyl et al., 2016) analyzed the internal mechanism of temperature s influence on emotion from the physiological level. They found that weather regulates mood by affecting the brain’s serotonin and central dopamine systems. Warmer temperature increase serotonin levels, while boosting dopamine production, making people feel more positive emotional and mental energy. The above studies have shown that there is a certain correlation between temperature and mood.

In this study, we focused on the correlation between physiological emotions and three different indoor temperatures, namely hot, comfort and cold. We established an emotion dataset of the Galvanic Skin Response (GSR) physiological signals under indoor temperature. We selected 25 videos describing five different emotional states (i.e., happy, angry, fear, sad, and calm) as eliciting material. In different indoor temperature, subjects watched inspirational video and recorded the subjects’ skin resistance data at the same time. The contents of the database are shown in Table 1. Moreover, we analyzed the physiological emotion and explore the correlation between indoor temperature and physiological emotions，using SVM, LSTM and ACRNN models. The major contribution of this article can be summarized as follows: (1) we develop a novel GSR physiological signal database considering indoor temperature. (2) we adopt three models to recognize emotions on the database. (3) we explore the correlation between different indoor temperature and emotions. This article is organized as follows. In Section 2, we give an overview of existing physiological databases and the models used in this paper. Section 3 describes the establishment of the physiological signal database under indoor temperature in detail. Section 4 introduces the processing of physiological signals. Section 5 presents the experimental results and discusses the correlation between indoor temperature and emotion. Section 6 presents conclusions and future work.

**Table 1:** Summary of the database content.

Elicitation material	
Number of videos	25
Video source	Film clips, Video-sharing Platforms
Video length	60s
Number of temperature	3
Type of temperature	Hot temperature, comfort temperature, cold temperature
Self-report	Pleasure, arousal, and dominance
Subjects	
Number of subjects	25
Emotion categories	Joy, anger, fear, sad, and calm
Recorded signals	Galvanic skin response, at a sampling rate of 50 HZ

## 2. Related works

### 2.1. Physiological emotion databases

Researchers use different incentive sources to stimulate emotions to build a database of physiological signals. We summarize these databases in Table 2. According to the survey, emotional stimuli are generally divided into three types, namely pictures, music and videos. Studies (Plass-Oude Bos et al., 2006; Savran et al., 2006; Brown et al., 2011; Petrantonakis and Hadjileontiadis, 2011) use the International Affective Picture System (IAPS) to stimulate the different emotions of test subjects. The advantage of using pictures as emotion-evoking material is that the picture material is easy to obtain, but this method cannot continuously stimulate the tester’s emotions. Another studies (Lin et al., 2010; Hadjidimitriou and Hadjileontiadis, 2012) use music to induce emotions, and it is also proved that music can continuously induce the emotions of test subjects to a certain extent. Other studies (Koelstra et al., 2012; Soleymani et al., 2012; Zheng and Lu, 2015; Katsigiannis and Ramzan, 2018; Li et al., 2018; Song et al., 2019) adopt the pictures, music, and videos as emotion-inducing materials. The experimental results show that videos can induce the emotions of test subjects for a long time. Therefore, using video as a stimulus material to induce emotions is popular. Although recent research studied physiological emotion elicited by pictures, music, and videos, but they did not consider the impact of temperature on emotions. Therefore, it is necessary to establish a database of physiological signals under different indoor temperature conditions and analyze the emotion between temperature and emotion.

**Table 2:** Physilogical emotion database summary table.

Database	Stimuli	Sub	Feature extraction method and classifier	Emotional states	Emotional model
ENTERFACE/06 (Savran et al., 2006)	IAPS	5	STFT features and TBM	Positive, negative and neutral	Discrete model
Plass-Oude Bos et al. (2006)	IAPS,IADS	5	Fourier analysis, PCA and FDA	Valence and arousal	Dimensional model
Petrantonakis and Hadjileontiadis (2011)	IAPS	16	Asymmetry index of alpha and beta power, SVM	Positive and negative	Dimensional model
Brown et al. (2011)	IAPS	11	Spectral power features, KNN	Positive, negative and neutral	Dimensional model
Lin et al. (2010)	Music	26	PSD, DASM, RASM features, SVM and MLP	Joy, anger, sadness, pleasure	Discrete model
Hadjidimitriou and Hadjileontiadis (2012)	Music	9	SPG, HHS, ZAM features, KNN, QDA, and SVM	Liking and disliking	Discrete model
MAHNOB-HCI (Soleymani et al., 2012)	Video	27	Spectral power features, SVM and Adaboost	Valence and arousal	Dimensional model
DEAP (Koelstra et al., 2012)	Video	32	Spectral power features, Gaussion naïve Bayes classifier	Valence, arousal and liking	Dimensional model
SEED (Zheng and Lu, 2015)	Video	15	PSD, DE, DASM, RASM, DCAM features, SVM, KNN and DBN	Positive, negative and neutral	Discrete model
RCLS (Li et al., 2018)	Video	14	PSD, DE, HOC, FD, Wavelet, Hjorth features, GRSLR, GSCCA, GraphSC, CCA, SVM, and RF	Positive, negative, and neutral	Discrete model
DEAMER (Katsigiannis and Ramzan, 2018)	Video	23	PSD features, KNN, LDA, and SVM	Valence, arousal, and dominance	Dimensional model
MPED (Song et al., 2019)	Video	23	PSD, STFT, HHS, HOC, Hjorth features, KNN, and SVM	Joy, funny, disgust, anger, fear, sad, and neutrality	Discrete model
Our database	Video, temperature	25	SVM, LSTM, and ACRNN	Joy, anger, fear, sad, and calm	Discrete model

### 2.2. Methods for physiological emotion recognition

Classification models are used for emotion recognition from physiological signals. SVM is a powerful classification algorithm with the ability to handle data well. The core idea of SVM is to maximize the distance between the hyperplane and the shortest distance between samples of different categories, so as to realize binary classification or multi-classification tasks. As a special RNN, long short-term memory (LSTM) uses memory cells to deal with the problem of gradient disappearance and solve the problem of long-term time dependence. LSTM have been applied to solve many difficult problems such as speech emotion recognition (Atmaja and Akagi, 2019), facial expression recognition (Kabir et al., 2021), and text annotation (AlBatayha, 2021). Soleymani et al. (2011) applied LSTM to emotion recognition from physiological signals. Tao et al. (2020) proposed an attention-based convolutional recurrent neural network (ACRNN) for sentiment analysis and obtained good classification results. In this paper, we use SVM, LSTM, and ACRNN to evaluate the database.

## 3. Temperature-video physiological signal database

In order to study the influence of different indoor temperature on emotions, we designed three temperature, hot, comfortable and cold. We stimulated emotions through videos, collected the corresponding GSR emotional signals under these three temperature, and established a video-evoked physiological signal dataset under indoor temperature (VEPT).

### 3.1. Emotional model

Emotional model is critical when building the physiological signal database. At present, there are two kinds of emotion models that are widely used. One is the discrete emotion model and the other is the dimensional emotion model. Dimensional emotion models use two or more dimensions to define emotion. How to convert emotional states into two-dimensional or high-dimensional spatial locations and quantify emotional states in space is the main focus in the study of dimensional emotional models (Russell, 1980). The discrete emotion model refers to the idea that emotions are composed of basic emotions. Plutchik (2001) believes that the basic human emotions include fear, anger, happiness, sadness, surprise, disgust, tolerance, and anticipation. Ekman summarizes the characteristics of basic emotions and proposes six common basic emotions: joy, anger, sadness, fear, surprise, and disgust (Ekman, 1972). This discrete emotion model had a profound impact on human emotion research. In actual experiments, it was found that the emotions of surprise and disgust were difficult to stimulate, so we removed the emotions of surprise and disgust and added calm emotions on the basis of Ekman’s 6 basic emotions. This article uses these five emotions to build our VEPT database.

### 3.2. Video clip material

We selected 225 initial stimulus videos from video websites by retrieving emotional keywords, and edited them into 1-min segments. The video was viewed and assessed by 8 graduate student volunteers (4 males, 4 females). Volunteers watched these 225 videos sequentially. After watching the videos, the volunteers used discrete questionnaires to label the videos with emotion and give a 5-point rating to the videos according to their real feelings. According to the scoring results, the five videos with the highest ratings in each emotion are selected, and a total of 25 videos are used as the final emotional stimulation materials.

### 3.3. Temperature settings

Based on physiological signals, we induced subjects with different emotions in different indoor temperature by controlling temperature and variables. All the experiments were carried out in the laboratory. Three different temperatures are set: hot, comfortable, and cold. According to research (Cui et al., 2013; Barbosa Escobar et al., 2021), the hot temperature of the human body is between 30 and 40°C, the comfortable temperature is between 22 and 26°C, and the cold temperature is between 0 and 10°C. In this paper, the indoor temperature is controlled between 30 and 40°C to adapt to the hot temperature conditions, the room temperature is controlled between 22 and 26°C to adapt to the comfortable temperature conditions, and the room temperature is controlled at 0 and 10°C to adapt to cold temperature conditions. During collection, subjects were not disturbed by other external stimuli.

### 3.4. Physiological signals sampling

#### 3.4.1. Experimental equipment

Physiological signals were relatively weak electrical signals generated by the human body, and the equipment used for acquisition would affects the accuracy of the data. In this paper, the physiological signal acquisition equipment consisted of a self-customized skin sensor^1^ and a USB interface, which provided power to the instrument. The sampling frequency was 50 Hz. The skin resistance sensor adopts an external current elicitation method to measure the change signal of human skin resistance. For this reason, amplifiers were added to the sensor to make the output data more precise. There was a serial port debugging assistant to receive the data, which saved the data as a hexadecimal txt format file.

#### 3.4.2. Experimental subjects

The emotional changes of the experimental participants are the most important part of the collection process. Therefore, it is necessary that the test subjects are physically and mentally healthy, which ensures that they can watch the video carefully during the experiment. There were a total of 25 subjects in the experiment. Before the experiment, the subjects filled out a questionnaire containing personal information and ensured their physical health and the SCL-90 assessment scale for psychological evaluation, which was to ensure that their mental health was in good condition. Finally, the experimenter explained the experimental situation and obtained signed consent.

#### 3.4.3. Experimental process

**Figure 1:**
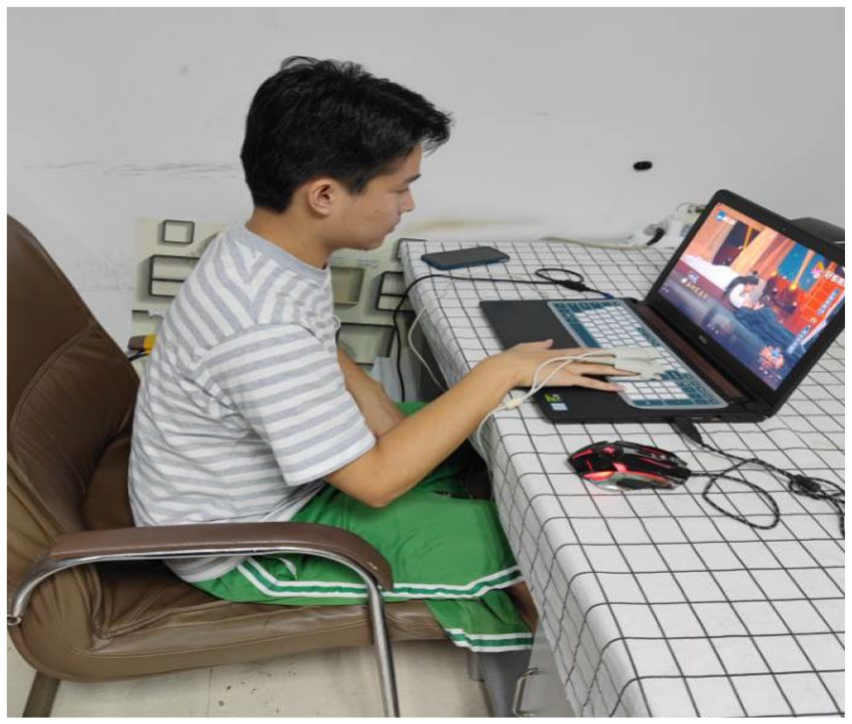
Example of use of collection equipment.

First, we let the subjects fully familiarize themselves with the laboratory temperature and they sit on the chair to adjust to a comfortable sitting position. The electrodes of the electrical skin sensor were placed on the index finger and middle finger of the subject’s left hand, and the palm was placed flat on the table, as shown in Figure 1. In order to ensure the accuracy of the collected data, the subjects were allowed to rest for 2 min before the experiment to ensure that they were in a calm state. At this time, five emotional videos were randomly selected from the motivational material, and subjects watched five emotional video clips sequentially in three temperature, and their skin resistance data were recorded. Then we played relaxing music to help them to rest for 2 min. After they calmed down, the next emotional video clip was displayed, until all emotion tests were over. Video clips were presented in the following order: joy, sadness, anger, fear, and calm. Finally, the skin electrical signals in different temperature were obtained.

## 4. Feature extraction from physiological signals

### 4.1. Data preprocessing

#### 4.1.1. Data denoising

**Figure 2:**
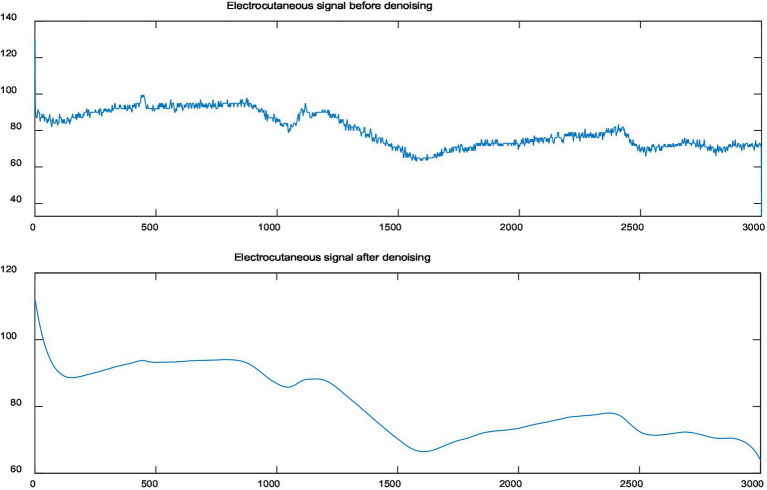
Galvanic skin response (GSR) signal comparison before and after denoising.

The galvanic skin signal is relatively weak, and it is easily affected by the noise of the acquisition instrument and the electromagnetic interference in the temperature during the acquisition process, so it is necessary to denoise the galvanic skin signal. The ButterWorthFilter is the simplest among the four classical filters. It can effectively remove the baseline and useless information and restore the signal. In this paper, the first-order ButterWorthFilter is used to denoise the GSR signal with the cut-off frequency set to 0.3 Hz. As shown in the Figure 2, the GSR signal is compared before and after denoising.

It can be clearly seen that the signal is more disturbed by noise before denoising, and the signal becomes smoother after denoising.

#### 4.1.2. Data normalization

There are great differences in different people’s skin electrical benchmarks, even in the same person in different states. Therefore, in order to accurately study the relationship between the electrical skin signal and emotional state, it is essential to eliminating individual differences in the signal. The specific operation is to subtract the data of a tester in the five emotional states from the mean value of the data in his corresponding calm state, so that the obtained data has removed individual differences, that is, normalized processing. The following formula, which ${\tilde{x}}_{calm}$ represents the mean value of the subject’s corresponding calm emotion, $x_{emotion}$ represents the 3,000 data points of a certain emotion of the tester, and $\tilde{x}$ is the normalized emotional data of the tester, which is also 3,000 data points after normalization.


(1) $$\tilde{x}=x_{emotion}-{\tilde{x}}_{calm}$$

### 4.2. Feature extraction

**Figure 3:**
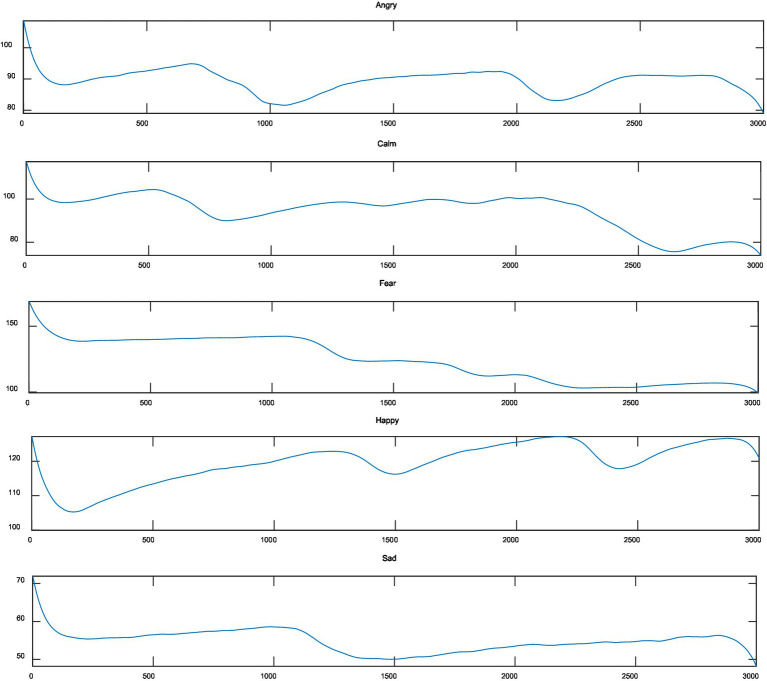
Galvanic skin response signals under five emotions.

After preprocessing the skin electrophysiological signal, the individual differences and noise interference of the skin electrophysiological response signal were removed, and then the data was analyzed to calculate the original characteristics of the signal. Figure 3 shows the skin electrophysiological signals collected by the testers in the five emotional states. From Figure 3, we can see that the skin electrophysiological levels are different in different emotional states. And the range of change is also different, especially the emotion of joy is the most obvious, followed by sadness. In the experiment, we can observe that when the same tester watches different emotional stimulation materials, the skin galvanic level changes obviously with the different emotional categories and strengths. Visually, it can be clearly seen that the electrodermal signal contains rich emotional change information in the time domain. Therefore, it is advisable to extract the statistical value of the galvanic skin response signal in the time domain as the feature value. Reference (Kim and André, 2006) in this paper uses a time window of 1 s to segment the original GSR signal after denoising and normalization. In each time window, the average value, standard deviation, first-order difference and second-order difference are, respectively, extracted as the features of emotion recognition.

## 5. Experimental results and discussion

### 5.1. Support vector machine

Support vector machine (SVM) (Liu and Guifang, 2021; Tuncer et al., 2021; Satyanarayana et al., 2022) is a kind of machine learning classification and regression algorithm, which is a typical representative of supervised machine learning model. The basic principle of SVM is to classify by maximizing the distance between the hyperplane and the closest distance to samples of different categories. For linearly separable binary classification problems, SVM finds an optimal hyperplane in the feature space, which maximizes the distance (i.e., interval) from the sample point closest to the hyperplane to the hyperplane. The sample points closest to the hyperplane (those on the dashed line) are called support vectors because they determine the location of the hyperplane. The goal of SVM is to minimize the number of misclassified points while maximizing the interval, so the optimization problem of SVM can be transformed into a quadratic programming problem for solution. If the data set is not linearly separable, the kernel function can be used to map the data into a high-dimensional space for classification.

### 5.2. Deep learning classification model for emotion recognition

#### 5.2.1. Long short term memory

The LSTM (Long Short Term Memory) (Du et al., 2020; Lew et al., 2020; Li et al., 2021) network is a neural network obtained by improving the traditional recurrent neural network (RNN). The innovation in LSTM is the introduction of the state of cell, which can be understood as a long-term memory. If the state is not modified, it will be passed on directly. But the memory will always change, so it is necessary to add some modules to modify the memory. Here is to add some concepts of “gates.” The gate is to allow some information to pass through, and some information is filtered by the gate. For example, a sigmoid layer is used to output a A vector with a value between 0 and 1. Multiply the vector and the cell state point by point (this is an information filtering operation). There are three such “gate” structures in lstm. Among them, the functions of the three gates of LSTM are: (1) Forgetting gate: determine how much information needs to be forgotten in the state of the memory unit at the last moment, and how much information is retained in the state of the current memory unit. (2) Input gate: control how much information of the input information candidate state at the current moment needs to be saved to the current memory unit state. (3) Output gate: Control how much information of the memory unit state at the current moment needs to be output to the external state.

#### 5.2.2. Attention-based convolutional recurrent neural network

**Figure 4:**
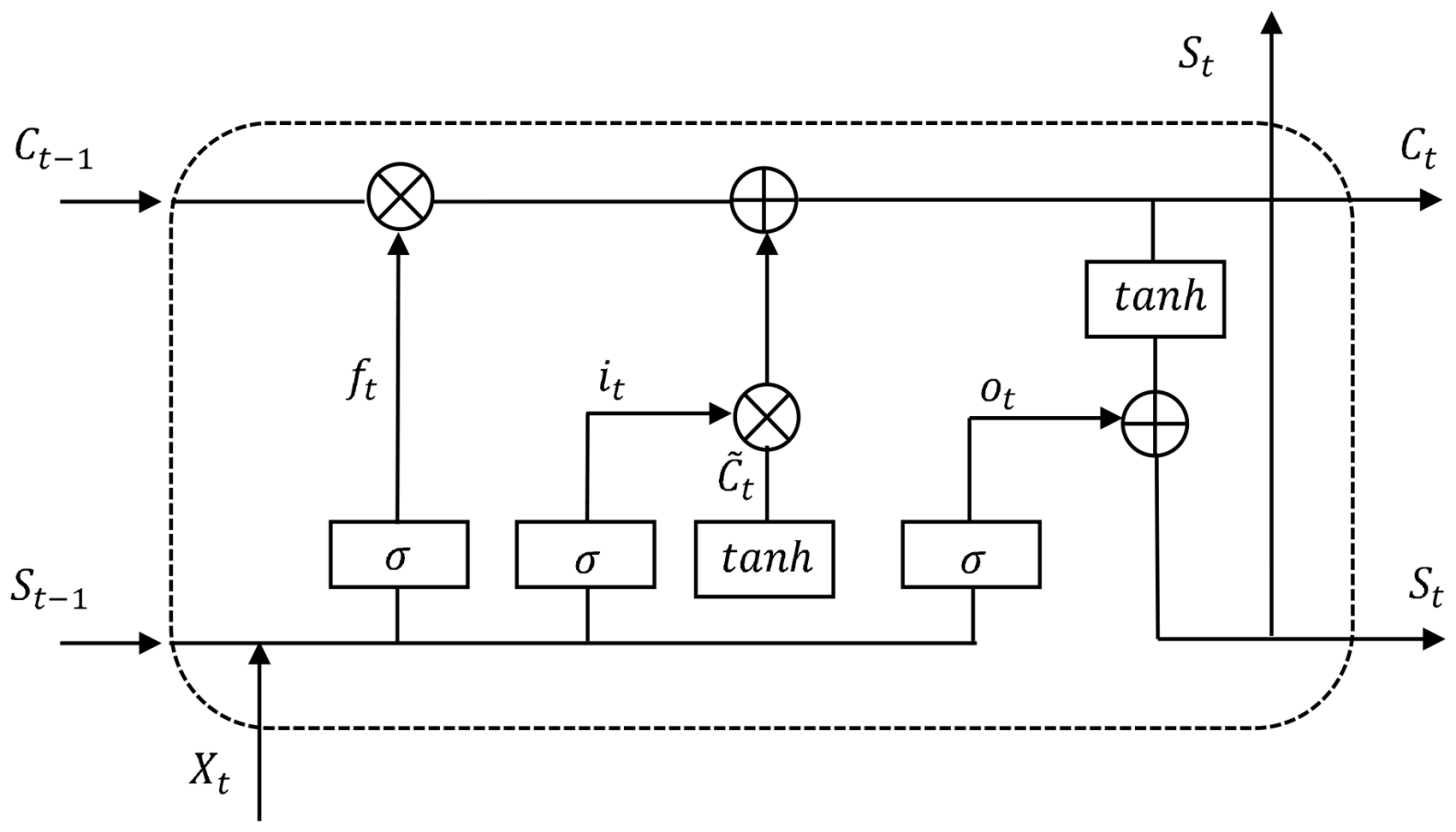
Long short-term memory (LSTM) unit architecture.


The ACRNN (Tao et al., 2020) network is inspired by Convolutional Recurrent Neural Networks (CRNN). Extract spatial information of GSR signals using CNN. Two LSTM layers are then used to extract temporal information, and an attention mechanism is integrated into the LSTM to explore the importance of different GSR samples, where the LSTM cell structure is shown in Figure 4. Among them, f_t_ is the forget gate, which is a vector, in which each element is between 0 and 1, which determines which features of C_(t-1)_ are used to calculate C_t_. C_(t-1)_ is the value of the memory cell at the previous time point, and X_t_ is the current input. f_t_ usually uses the sigmoid function as the activation function, and the calculation expression of f_t_ is as follows.

(2) $$f_{t}=\sigma \left(W_{f}\cdot \left[S_{t-1},X_{t}\right]+b_{f}\right)$$

i_t_ is the input gate, and like f_t_, it is also a vector, where each element is between 0 and 1, which is used to control which features of C_t_ are used to update C_t_. C_t_ is the cell state update value, calculated from X_t_ and C_(t-1)_. The sigmoid function is also used as the activation function here, and the calculation expression of i_t_ is as follows.


(3) $$i_{t}=\sigma \left(W_{i}\cdot \left[S_{t-1},X_{t}\right]+b_{i}\right)$$

(4) $${\tilde{C}}_{t}=\tanh\left(W_{c}\cdot \left[S_{t-1},X_{t}\right]+b_{C}\right)$$

(5) $$C_{t}=f_{t}{}^{*}C_{t-1}+i_{t}{}^{*}{\tilde{C}}_{t}$$

o_t_ is the output gate, which is also a vector to control which memory values in the memory unit are output. The sigmoid function is also used as the activation function here, and the calculation expression of o_t_ is as follows.


(6) $$o_{t}=\sigma \left(W_{o}\cdot \left[S_{t-1},X_{t}\right]+b_{o}\right)$$

Finally, the output S_t_ of the hidden node is calculated, which is calculated by the output gate o_t_ and the unit state C_t_. The calculation expression of S_t_ is as follows.


(7) $$S_{t}=o_{t}*\tanh\left(C_{t}\right)$$

Moreover, the attention mechanism can update the weights according to the similarity of GSR signals, so it can obtain more discriminative temporal features of GSR signals.

### 5.3. Comparison of different classifier

The GSR signals collected in a comfortable temperature are processed as the input of SVM, LSTM and ACRNN models, respectively, and the models are trained for five classifications. The data obtained after denoising and normalizing the GSR signal collected at a comfortable temperature are processed as the input of the SVM, LSTM and ACRNN models, respectively. After model training, five classifications are carried out. The five categories of emotions are: anger, fear, happy, calm, sad. The classification results of each model are shown in Table 3. This paper uses the skin resistance data for classification calculation. Traditional classification methods ignore the variation of skin resistance value in the time dimension, and LSTM, ACRNN models solve this problem. It can be seen from the table that the recognition accuracy of ACRNN, LSTM is higher than that of the SVM algorithm. By comparison, the effect of the ACRNN model is the best among the four classification methods. In this paper, we will use LSTM and ACRNN to perform emotion recognition on physiological signals in three indoor temperature, further discussing the correlation between temperature and emotion.

**Table 3:** Emotion recognition rate at comfortable temperature.

Emotion Model	Anger	Fear	Happy	Sad	Calm	Average
SVM	0.513	0.487	0.652	0.462	0.583	0.539
LSTM	0.525	0.574	0.724	0.509	0.695	0.605
ACRNN	0.634	0.648	0.845	0.610	0.769	0.701

### 5.4. Experimental results under three temperature

#### 5.4.1. Experimental results under comfortable temperature

**Figure 5:**
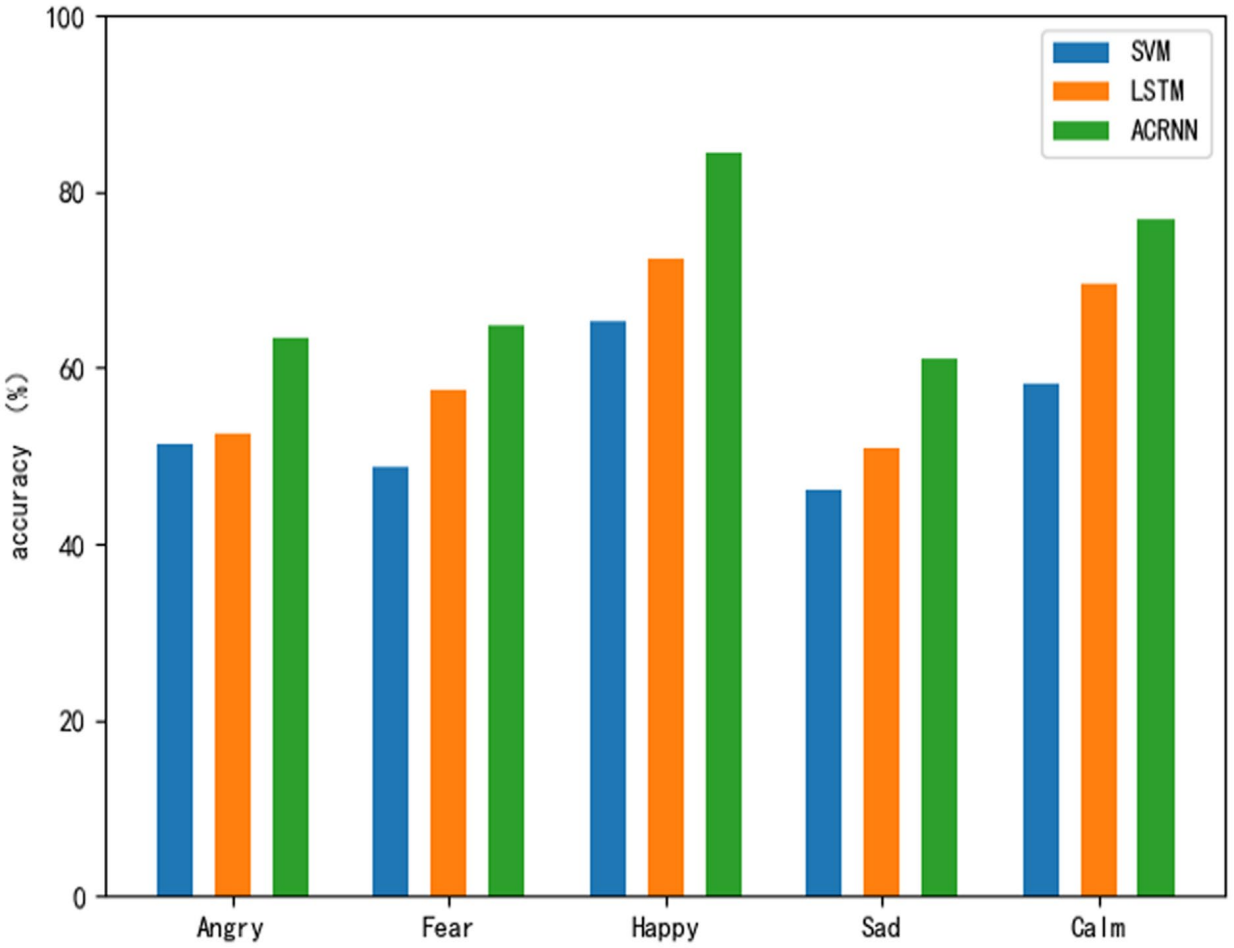
Sentiment classification results at comfort temperature.

Table 3 shows the emotion recognition results of SVM, LSTM and ACRNN at a comfortable temperature. From Figure 5, we can clearly see the emotion recognition results of the three models at a comfortable temperature. Judging from the classification results of each model alone, the emotion of joy is the emotion with the best classification effect in each model, followed by the emotion of calm. The emotions Fear and Anger were similarly recognized across the models, but not as good as Joy and Calm, while Sadness was the worst emotion among the models. From the overall perspective of the five emotions, it can also be seen that the recognition effect of joy is better than that of other emotions on the whole. Joy is more easily aroused than other emotions at a comfortable temperature. This shows that the amplitude of the electrodermal signal of joy emotion will increase at a comfortable temperature, and its corresponding time-domain features are more obvious than those of other emotions, and are easier to be recognized by the classification model. However, the three emotions of anger, fear, and sadness were less recognized than the other two emotions, which indicated that the characteristics of these three emotions were not as obvious as joy at the comfort temperature.

#### 5.4.2. Experimental results under hot temperature

**Figure 6:**
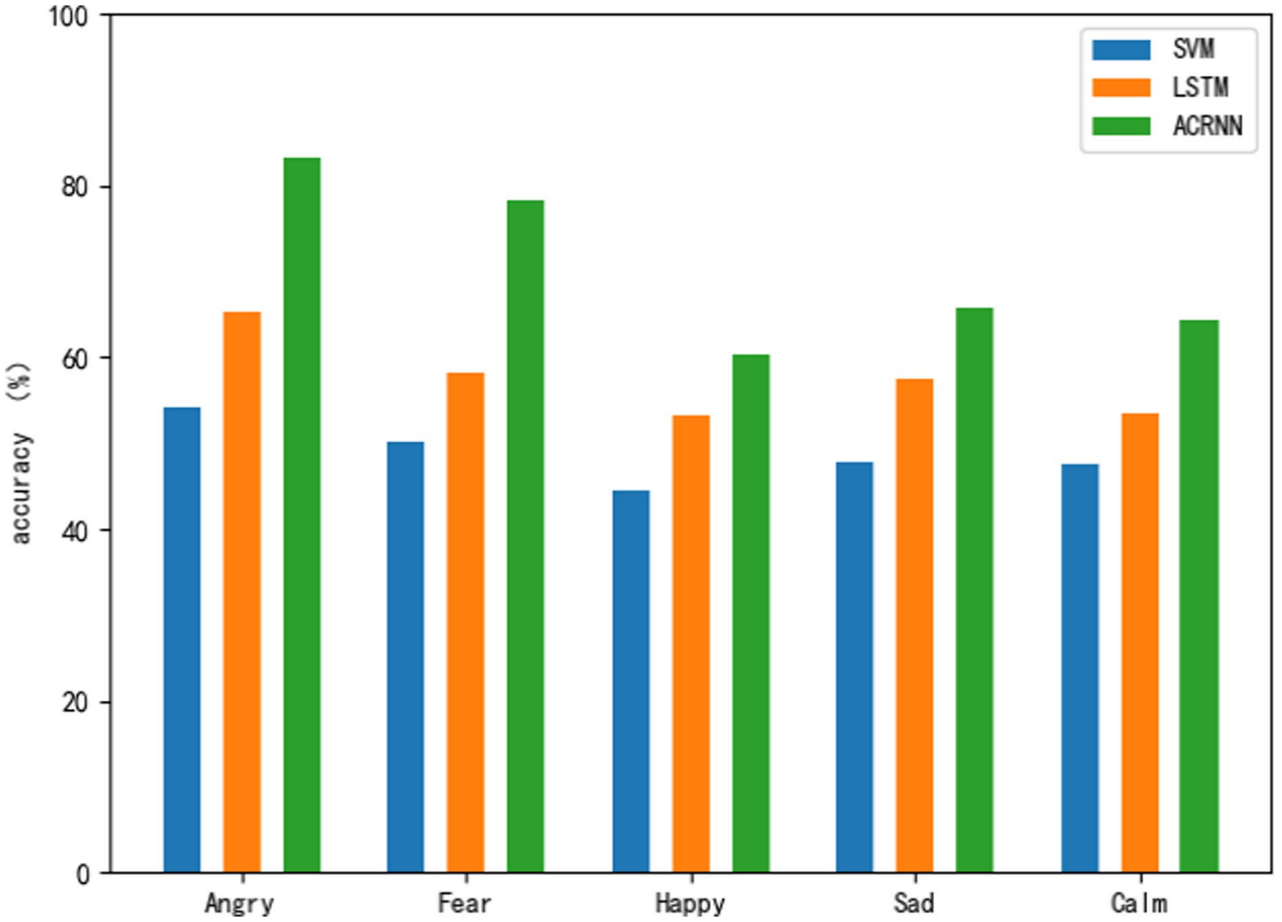
Sentiment classification results at hot temperature.

Use three classification models to perform emotion recognition on five emotions under hot temperature, and analyze the influence of hot temperature on various emotions according to the classification results in Table 4. From Figure 6, we can clearly see the emotion recognition results of the three models at hot temperature. Also from the classification results of each model, the two emotions of anger and fear are the emotions with the best classification effect in each model, and the classification effect of calmness, joy and anger in each model is not as good as the other two emotions. Emotion. Looking at the five emotions as a whole, it can also be seen that anger and fear are generally better recognized than the other three emotions, which shows that the characteristics of anger and fear are more obvious than other emotions at hot temperatures.

**Table 4:** Emotion recognition rate at hot temperature.

Emotion model	Anger	Fear	Happy	Sad	Calm	Average
SVM	0.541	0.501	0.445	0.478	0.476	0.488
LSTM	0.653	0.581	0.533	0.574	0.534	0.575
ACRNN	0.832	0.782	0.603	0.657	0.644	0.704

#### 5.4.3. Experimental results under cold temperature

**Figure 7:**
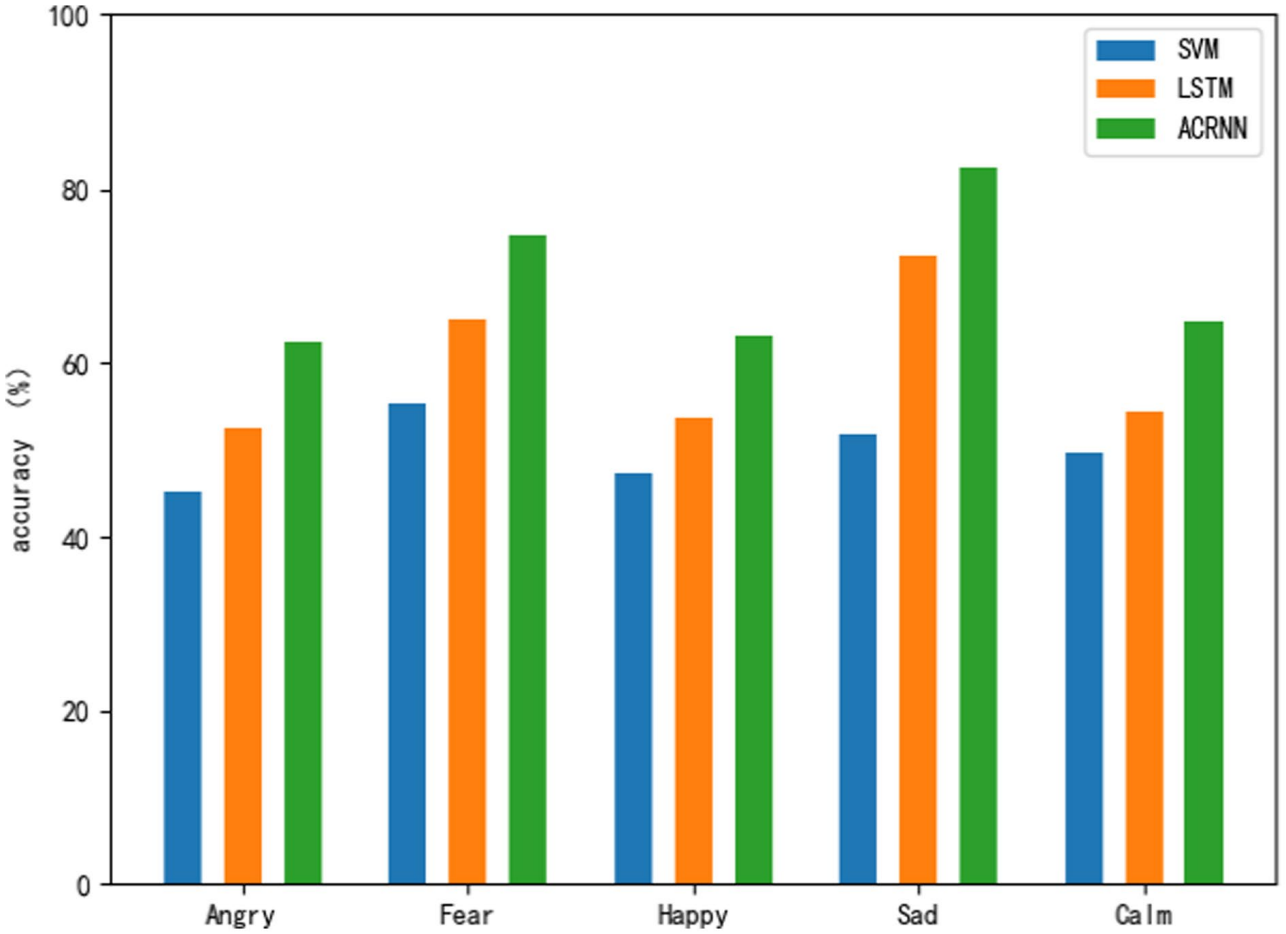
Sentiment classification results at cold temperature.

Use three classification models to carry out emotion recognition on five emotions under cold temperature, and analyze the influence of cold temperature on various emotions according to the classification results in Table 5. As shown in Figure 7, the emotion recognition results of the three models at cold temperatures can be clearly seen. Judging from the classification results of each model alone, the two emotions of sadness and fear are the emotions with the best classification effect in each model. Followed by calm, joy and anger, the two emotions are the worst emotions in each model. Looking at the five emotions as a whole, it can also be seen that the recognition effect of sadness and fear is higher than that of the other three emotions.

**Table 5:** Emotion recognition rate at cold temperature.

Emotion model	Anger	Fear	Happy	Sad	Calm	Average
SVM	0.452	0.553	0.472	0.519	0.496	0.498
LSTM	0.526	0.651	0.537	0.723	0.545	0.596
ACRNN	0.624	0.748	0.631	0.826	0.648	0.695

#### 5.4.4. Comparison of emotion recognition results at three temperatures

**Figure 8:**
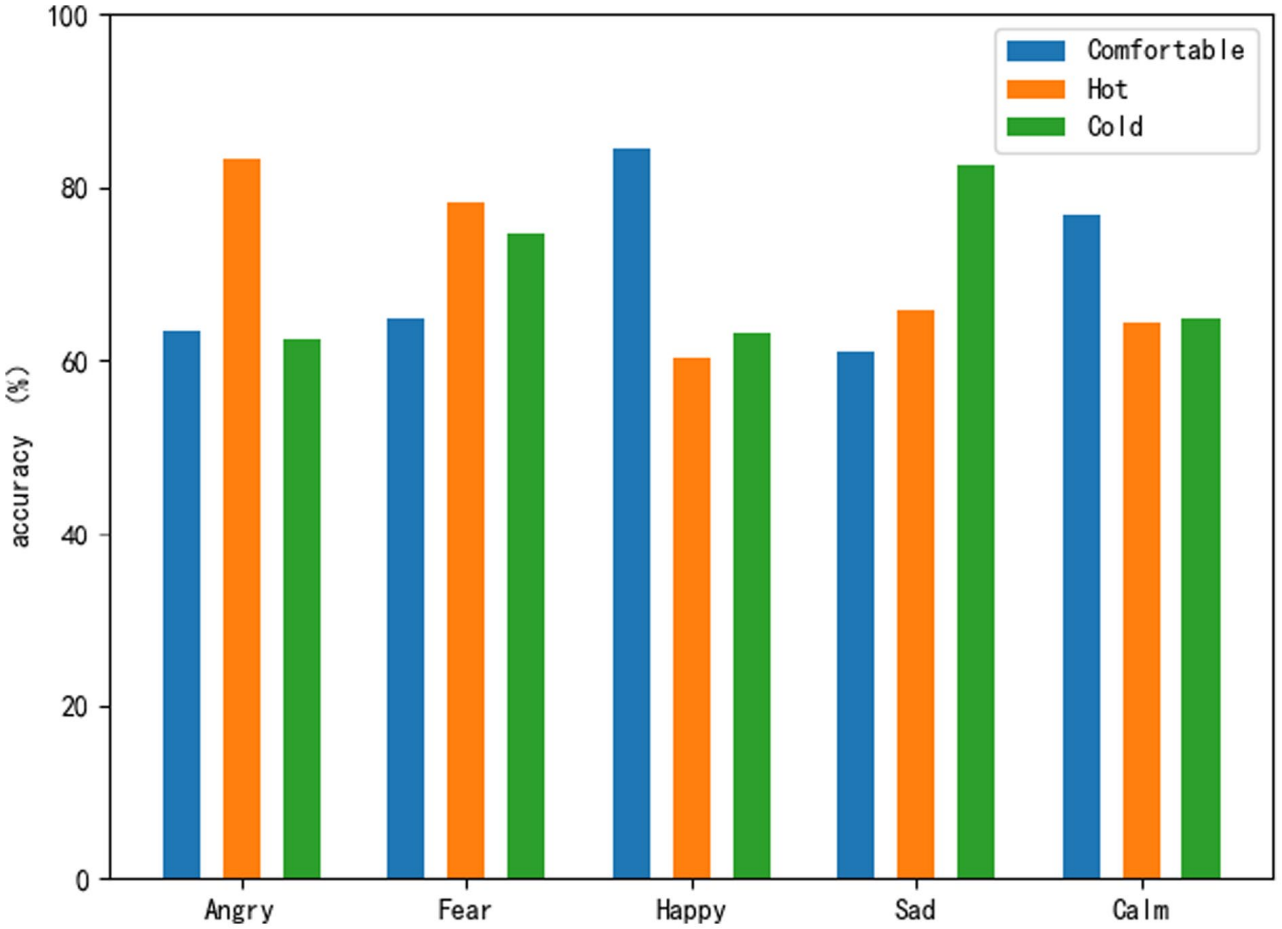
Sentiment classification recognition rate of ACRNN under three temperatures.

In this section, based on the ACRNN model with the best recognition effect, this paper draws and compares the results of physiological signal emotion recognition at three temperatures, and analyzes the impact of different temperatures on human emotions based on the classification results at three temperatures. Figure 8 shows the recognition rate of five emotion classifications by ACRNN at three different temperatures. Comparing the five emotions at the comfortable temperature and the hot temperature can clearly see the recognition effect. Compared with the comfortable temperature, the recognition rate of the two emotions anger and fear is improved at the hot temperature. The recognition rates of joy and calm were lower in hot temperature than in comfort temperature. Comparing the five emotions at the comfortable temperature and the cold temperature, it can be seen that the recognition rate of the two emotions of sadness and fear is improved in the cold temperature compared with the comfortable temperature. However, the recognition effect of joy and anger was reduced in the cold temperature compared with the comfortable temperature. Compared with the other two temperatures, the overall recognition rate of joy and calm was relatively better at the comfortable temperature. In general, compared with high temperature and cold temperature, positive emotion recognition effect is relatively better in comfortable temperature. Compared with the comfortable temperature, the effect of negative emotion recognition in hot and cold temperatures is relatively better. This suggests that comfortable temperature promotes positive emotions to a certain extent, while hot and cold temperatures promote negative emotions to a certain extent.

## 6. Conclusion and future work

In this paper, a physiological signal emotion database including three indoor temperatures is established. Three classification models are used to recognize the emotion of GSR signals in three temperatures, and the recognition results are compared and analyzed. The experimental results showed that positive emotions were promoted in a comfortable temperature, while negative emotions were promoted in a hot and cold temperature. Studies have found that indoor temperature has a certain impact on people’s positive and negative emotions. There are still some shortcomings in this experiment. The age group of the subjects is relatively single and the number is limited. In future experiments, the range of subjects should be expanded to improve the overall effect of the experiment.

## Data availability statement

The raw data supporting the conclusions of this article will be made available by the authors, without undue reservation.

## Ethics statement

Ethical review and approval was not required for the study on human participants in accordance with the local legislation and institutional requirements. The patients/participants provided their written informed consent to participate in this study. Written informed consent was obtained from the individual(s) for the publication of any potentially identifiable images or data included in this article.

## Author contributions

KW: design, methodology, and writing – reviewing. ZZ: data collection, software, and writing – original draft preparation. XS: investigation. KW and TY: writing – editing. All authors contributed to the article and approved the submitted version.

## Funding

This work was supported by the National Natural Science Foundation of China (grant no. 62001004), the Academic support project for top-notch talents in disciplines (majors) of colleges and universities in Anhui Province (gxbjZD2021067), the Research and development fund of Anhui University of Architecture (JZ202118), and the open project of Anhui Key Laboratory of building acoustic environment, Anhui University of Architecture (AAE2021ZR02).

## Conflict of interest

The authors declare that the research was conducted in the absence of any commercial or financial relationships that could be construed as a potential conflict of interest.

## Publisher’s note

All claims expressed in this article are solely those of the authors and do not necessarily represent those of their affiliated organizations, or those of the publisher, the editors and the reviewers. Any product that may be evaluated in this article, or claim that may be made by its manufacturer, is not guaranteed or endorsed by the publisher.

## Footnotes

^1^ http://www.hfhuake.com
